# Maternal health care service utilization among young married women in India, 1992–2016: trends and determinants

**DOI:** 10.1186/s12884-021-03607-w

**Published:** 2021-02-10

**Authors:** Pooja Singh, Kaushalendra Kumar Singh, Pragya Singh

**Affiliations:** Department of Statistics, Institute of Science, Banaras Hindu University, Varanasi, Uttar Pradesh 221005 India

**Keywords:** Maternal health care, Antenatal care, Skilled birth attendance, Young women, India, NFHS, Pooled data

## Abstract

**Background:**

Maternal deaths among young women (15–24 years) shares 38% of total maternal mortality in India. Utilizing maternal health care services can reduce a substantial proportion of maternal mortality. However, there is a paucity of studies focusing on young women in this context. This paper, therefore, aimed to examine the trends and determinants of full antenatal care (ANC) and skilled birth attendance (SBA) utilization among young married women in India.

**Methods:**

The study analysed data from the four rounds of National Family Health Surveys conducted in India during the years 1992–93, 1998–99, 2005–06 and 2015–16. Young married women aged 15–24 years with at least one live birth in the 3 years preceding the survey were considered for analysis in each survey round. We used descriptive statistics to assess the prevalence and trends in full ANC and SBA use. Pooled multivariate logistic regression was conducted to identify the demographic and socioeconomic determinants of the selected maternity care services. The significance level for all analyses was set at *p* ≤ 0.05.

**Results:**

The use of full ANC among young mothers increased from 27 to 46% in India, and from 9 to 28% in EAG (Empowered Action Group) states during 1992–2016. SBA utilization was 88 and 83% during 2015–16 by showing an increment of 20 and 50% since 1992 in India and EAG states, respectively. Findings from multivariate analysis revealed a significant difference in the use of selected maternal health care services by maternal age, residence, education, birth order and wealth quintile. Additionally, Muslim women, women belonging to scheduled caste (SC)/ scheduled tribe (ST) social group, and women unexposed to mass media were less likely to utilize both the maternal health care services. Concerning the time effect, the odds of the utilization of full ANC and SBA among young women was found to increase over time.

**Conclusions:**

In India coverage of full ANC among young mothers remained unacceptably low, with a wide and persistent gap in utilization between EAG and non-EAG states since 1992. Targeted health policies should be designed to address low coverage of ANC and SBA among underprivileged young mothers and increased efforts should be made to ensure effective implementation of ongoing programs, especially in EAG states.

## Background

Although global maternal mortality declined by 38% between 2000 and 2017, it remains unacceptably high with nearly 810 women dying every day during and following pregnancy and childbearing [1]. A vast gap in maternal mortality ratios (MMR) still exists between rich and poor countries as 99% of global maternal deaths occurred in low- and middle-income countries (LMICs) [2]. In particular, India had 35,000 maternal deaths in 2017 which is 12% of the global share [1]. MMR of India has come down to 122 per 100,000 live births in 2015–2017. However, there is still a long way to attain the target mentioned in the Sustainable Development Goals (SDGs), particularly in Empowered Action Group (EAG) states as they contribute to the maximum maternal deaths with MMR being 175 per 100,000 live births [3]. Moreover, of all maternal deaths in India, around 38% occurred between the age group of 15–24 years during 2015–17 [3].

It is well documented that the use of maternal health care services, specifically antenatal care (ANC) during pregnancy and skilled attendance during delivery, plays a significant role in reducing maternal deaths [4–7]. The main purpose of ANC is the prevention and early diagnosis of pregnancy complications. It also serves as a counselling platform to improve the understanding of women and her family about the pregnancy, childbirth, and care of the new-born [8].

Likewise, skilled birth attendance (SBA) provides basic and emergency care to women during labour, delivery and the postpartum period [9]. The use of SBA insures delivery in presence of an accredited and skilled health professional trained to proficiently manage normal (uncomplicated) pregnancies, childbirth and the immediate postnatal period, and handle the identification, management and referral of complications in women and new-borns [9].

The recent National Family Health Survey (NFHS-4), 2015–16 estimated that only 51% of mothers attended the recommended number of four ANC visits, and 81% of mothers received SBA [10]. This elucidates that the scenario of ANC services is still very inadequate in India. Although the overall coverage of SBA is high, there is a significant disparity between rural-urban residents and among different states of India [11].

Several studies conducted in India have reported a significant association between women’s use of maternal health care services and different socio-demographic factors such as rural-urban residence, geographical region, educational level, ethnic group, religion, wealth index, parity/birth order, etc. [12–16]. However, most of these studies included all women of reproductive age (15–49 years) in their analysis. To the best of our knowledge, no study has explored the usage of these services among young women aged 15 to 24 years by utilizing the most recent NFHS data.

The United Nations defines ‘youth’ as persons between the ages of 15 and 24 years [17], and existing evidence shows that mothers of this age group are more susceptible to health/social challenges such as unwanted pregnancies, abortions, and sexually transmitted diseases (including HIV/AIDS) [18–20]. Furthermore, mothers of this age group (adolescents and adult young mothers) have an increased risk of developing serious obstetric complications and subsequent death during childbearing [21, 22]. It is estimated that, of all deaths globally in women aged 10–25 years, 15% are due to maternal causes [23].

Young people form a significant proportion (19.1% i.e. 231 million) of the Indian population and more than half of all births (54%) took place during this period [10, 24]. Furthermore, the incidence of early marriage and motherhood is widespread in India. According to NFHS-4, on average women in India have 1.5 children by the time they complete 24 years of age, and 27% of women aged 20–24 years were married before the age of 18 years [10]. Early marriage is also associated with lower uptake of maternity care services [25, 26]. Understanding the status of maternal healthcare service utilization among such a large and vulnerable portion of the population can provide meaningful evidence for the development of effective and targeted health care programs.

The present study, therefore, aimed to explore the prevalence, trends and determinants of maternal health care service (ANC and SBA) utilization among young married women in India using pooled data from four rounds of NFHS (1992–2016). This study only included married young women, since NFHS-1 (1992–93) and NFHS-2 (1998–99) did not collect information from unmarried women [27, 28]. Moreover, childbearing outside marriage is rare (0.3% during 2015–16) in India [10].

## Methods

### Data

The study utilized data from the four rounds of National Family Health Survey (NFHS) conducted during the years 1992–93 (NFHS-1), 1998–99 (NFHS-2), 2005–06 (NFHS-3) and 2015–16 (NFHS-4). NFHS is the Indian version of the Demographic Health Survey (DHS). It is a nationally representative, cross-sectional household survey conducted by the International Institute for Population Science (IIPS) under the guidance of the Ministry of Health and Family Welfare (MoHFW), Government of India. The survey covers a representative sample of women in the age group 15–49 years and provides reliable estimates of fertility, family planning practices, reproductive health, maternal and child health care, utilization and quality of health and family planning services and other related indicators across all the states/union territories and India as a whole. The number of women interviewed was 89,777 in NFHS-1, 91,000 in NFHS-2, 124,385 in NFHS-3 and 699,686 in NFHS-4 with the overall response rate of 96.1, 95.5, 94.5 and 96.7%, respectively [10, 27–29]. In this study, data of the currently married young women aged 15–24 years were considered for analysis in each round. The final analytic sample size (number of women that met our inclusion criteria) from the four survey rounds were 17,335, 14,243, 16,047 and 63,351 in NFHS-1, NFHS-2, NFHS-3 and NFHS-4 respectively. There was a fairly significant jump in the number of women interviewed from NFHS-3 to NFHS-4 to produce reliable indicator estimates for each district and for urban and rural areas in districts, which reflected in our final analytic sample size also. Information on the indicators of maternal health care services has been collected for different reference periods in the different rounds of NFHS. For the purpose of retaining consistency, the sample for this study was confined to the information for the last live birth in the 3 years preceding the date of survey. There are 36 states/union territories in India. The samples from two states, namely Sikkim and Tripura, were excluded from the final analytic samples as the required information in states of Sikkim and Tripura were missing in NFHS-1 and NFHS-2, respectively. Appropriate sample weights were used taking cognizance of the survey design to make the estimates representative and comparable over all the survey rounds. A detailed description of the survey design including sampling weights and other information is provided in the round-specific reports [10, 27–29].

### Outcome variables

The study used two indicators to measure the utilization of maternal health care services among young married women; ANC and SBA. Keeping in mind the World Health Organization’s recommendation and availability of information across all the four survey rounds, full ANC indicator was measured as those women who had four or more antenatal check-ups, had at least one tetanus toxoid injection and consumed iron and folic acid tablets or syrup for the last live birth during the 3 years preceding the survey period [10]. The SBA indicator includes those women who had their deliveries conducted either in public or private hospitals/health centres/clinics or at home assisted by trained health personnel (doctor/nurse/lady health visitor (LHV)/auxiliary nurse midwives (ANM)) [9]. Both the outcome indicators were measured with binary responses (used full ANC/SBA =1; otherwise = 0).

### Independent variables

Demographic and socioeconomic characteristics of women included as independent variables in the analysis were type of residence, state-wise residence, religion, social group, educational level, wealth quintile, maternal age, birth order, and mass media exposure. Survey round was included as a factor for analysing the use of maternal health care services among young women over time. These variables were selected because they have been found as significant predictors of maternal healthcare service utilisation in the literature [12–16, 30] The sub-categories of these variables are presented in Table 1.

**Table 1:** Description of independent variables

Variable	Categories
Type of residence	Rural and Urban
State-wise residence	EAG states and Other states (non-EAG states)^a^
Religion	Hindu, Muslim and Others (Christians, Sikhs, Buddhists, Jain, Jewish, Parsi, and others)
Social group	Scheduled Caste (ST), Scheduled Tribe (ST) and Others (non-SC/ST)^b^
Maternal Education	No education, Primary, Secondary and Higher
Wealth Quintile	Poorest, Poorer, Middle, Richer and Richest
Maternal age (in completed years)	≤19 years and 20–24 years
Birth order	1, 2 and 3+ (birth order three and above)
Mass media exposure	No exposure (accesses none of the three modes of mass media namely newspaper/magazine, radio, television/cinema) and Any exposure (access to any of the three media)
Survey Round	NFHS-1, NFHS-2, NFHS-3 and NFHS-4

^a^The MoHFW, India, established EAG in 2001 to have special focus by monitoring and facilitating the attainment of national health goals on 8 states (Bihar, Jharkhand, Madhya Pradesh, Chhattisgarh, Uttar Pradesh, Uttarakhand, Odisha, and Rajasthan) which are demographically lagging behind

^b^The Scheduled Caste (SCs) and Scheduled Tribes (STs) are officially designated groups of disadvantaged people in India

### Statistical analysis

Descriptive statistics were obtained for the demographic and socioeconomic characteristics of women aged 15–24 years who had a child in the 3 years preceding the surveys. The trends in the prevalence of full ANC and SBA utilization was analysed using weighted frequency percentages, stratified by the selected background characteristics. The trend was examined separately for the survey periods NFHS-1 to NFHS-2 (1992–98), NFHS-2 to NFHS-3 (1999–2005), NFHS-3 to NFHS-4 (2006–16), and NFHS-1 to NFHS-4 (1992–2016) to observe the changes over time.

Owing to the comparable sampling design of NFHS [31, 32], we have pooled all the four rounds of NFHS datasets to observe changes in maternal health care utilization among young married women over time. Since both the outcome indicators used in this study were dichotomous variables, pooled logistic regression models were fitted to assess the influence of demographic and socioeconomic predictors on the likelihood of utilizing full ANC and SBA**.** Before including in regression analysis, all the predictor variables were verified for association with outcome variables at the bivariate level using chi-square tests. We considered *p* ≤ 0.05 as the criterion for statistical significance. The results of the regression analysis have been presented as odds ratios (OR) with their 95% confidence intervals (CI). The entire analysis has been carried out using STATA version 14 (Stata Corporation, College Station, TX, USA) with svyset (SVY) commands to take into account the survey design (i.e. sampling weights with clustering and strata).

## Results

### Trends in sample characteristics

Table 2 presents the weighted percentage distribution of the study sample for each survey round by selected background characteristics. More than three-quarters of the young mothers were from rural areas across all four surveys. According to the NFHS-4, nearly 47% of the young mothers were residents of EAG states, and this percentage was similar in the earlier three surveys. The majority of women were Hindu (80–81%) and from ‘non-SC/ST’ social group (67–77%) in all four rounds of the survey. The distribution of women by wealth quintile remained more or less similar across all four surveys; around 22% in poorest, 24% in poorer, 23% in middle, 20% in richer and 12% in richest. On average, about half of the young women had no education in the first three surveys (62% in NFHS-1, 50% in NFHS-2, and 43% in NFHS-3), while in NFHS-4, 19% reported no education. The proportion of women with secondary education rose from 21% in NFHS-1 to 58% in NFHS-4. The percentage of young married women who had their last birth during their teenage declined from 44% in NFHS-1 to 32% in NFHS-4. The proportion of young women with first birth order increased from 48 to 58% while women with birth order three and above reduced from 22 to 9%. Exposure to any type of mass media among young mothers increased from 48% in NFHS-1 to 70% in NFHS-4.

**Table 2:** Weighted percentage distribution of women aged 15–24 years who had given birth in the 3 years preceding the survey by analysis variables and survey round

Variables	NFHS-1	NFHS-2	NFHS-3	NFHS-4	Total ^**a**^
Number	17,335	14,243	16,047	63,351	110,976
**Type of residence**					
Rural	79.37	79.67	76.36	75.09	76.53
Urban	20.63	20.33	23.64	24.91	23.47
**State-wise residence**					
EAG states	45.48	46.74	49.84	47.50	47.43
Other states	54.52	53.26	50.16	52.50	52.57
**Religion**					
Hindu	81.39	81.30	80.64	80.02	80.49
Muslim	14.09	14.66	15.52	15.85	15.37
Others	4.52	4.03	3.83	4.13	4.13
**Social group**					
Scheduled caste	13.51	20.44	21.28	22.14	20.45
Scheduled tribe	9.85	9.75	9.32	11.19	10.53
Others	76.63	69.81	69.39	66.67	69.02
**Educational level**					
No Education	62.05	50.42	41.92	19.31	33.25
Primary	15.96	17.13	16.64	14.35	15.29
Secondary	20.62	26.36	38.63	58.22	45.43
Higher	1.37	6.09	2.80	8.12	6.03
**Wealth Quintile**					
Poorest	20.40	21.25	22.49	21.66	21.53
Poorer	23.17	23.47	23.55	24.62	24.09
Middle	22.54	22.22	22.07	23.01	22.70
Richer	20.21	19.95	19.73	19.40	19.64
Richest	13.69	13.11	12.14	11.32	12.04
**Maternal age**					
≤ 19	44.46	43.20	42.07	31.77	36.71
20–24	55.54	56.80	57.93	68.23	63.29
**Birth order**					
1	44.78	44.69	47.14	57.78	52.53
2	32.68	32.91	34.47	33.28	33.31
3+	22.54	22.40	18.39	8.94	14.16
**Mass media exposure**					
No exposure	51.75	44.03	28.02	21.91	30.29
Any exposure	48.25	55.97	71.98	78.09	69.71

^a^percentage of total

### Trends in the utilization of maternal health care services

**Fig. 1:**
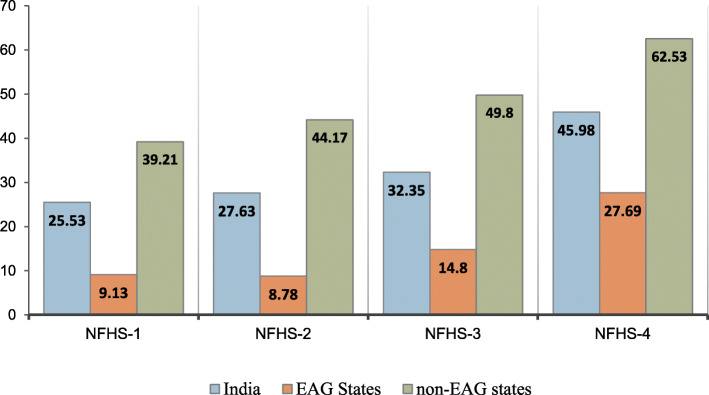
Trends in the use of full antenatal care among young married women in India and EAG states, 1992–2016

**Fig. 2:**
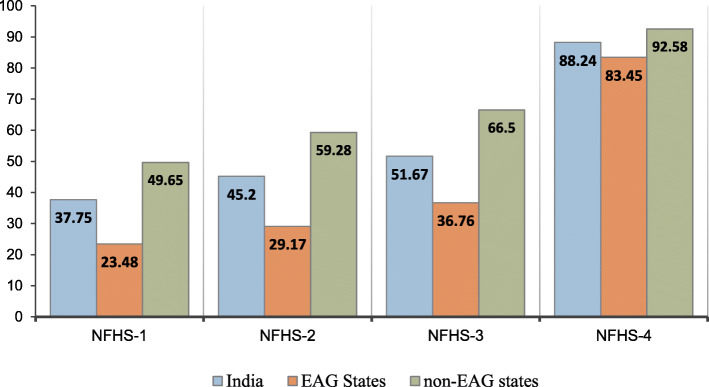
Trends in the use of skilled birth attendance among young married women in India and EAG states, 1992–2016

Figures 1 and 2 depict the trends in the use of full ANC and SBA, respectively, among young married women in India and EAG states during the period 1992–2016. The percentage of women who utilized full ANC increased from 25% in NFHS-1 to 45% in NFHS-4 in India, whereas in EAG states it was 28% in NFHS-4 with an increment of more than 3 times since NFHS-1. The proportion of women who had SBA rose from 38% in NFHS-1 to 88% in NFHS-4 in India, whereas in EAG states it increased from 23% in NFHS-1 to 83% in NFHS-4.

### Trends in the prevalence of full ANC utilization by background characteristics

Among urban residents, the largest increment of 7 percentage points (from 42 to 49%) in the use of full ANC was observed during 1992–98, whereas, among rural residents, the highest absolute change was observed during 2006–16 with 16% increase (from 26 to 46%). Around 27% of young mothers residing in non-EAG states reported having full ANC in NFHS-4, up from 9% in NFHS-1. During NFHS-1, 15% of ST young women had full ANC and the percentage rose to 43% during NFHS-4. Regarding education, young women with higher education showed greatest prevalence of full ANC utilization throughout the study period while the largest increment was observed among women with no education with a change of 12 percentage points. The proportion of young mothers belonging to the poorest wealth quintile who utilized full ANC has been increased from 10 to 27% between 1992 and 2016. The proportion of adolescent mothers who availed themselves of full ANC doubled; from 23% in NFHS-1 to 46.5% in NFHS-4. Women with first birth order showed the greatest prevalence and increment (30 to 44%) in the use of full ANC over the study period. (Table 3).

**Table 3:** Percentage of young married women who utilized full antenatal care by demographic and socioeconomic characteristics, India, 1990–2016

Variables	Prevalence (%) of full ANC utilization					Percentage change			
	NFHS-1	NFHS-2	NFHS-3	NFHS-4	Total ^a^	NFHS-1 to NFHS-2	NFHS-2 to NFHS-3	NFHS-3 to NFHS-4	NFHS-1 to NFHS-4
**Type of residence**									
Rural	21.23	22.19	26.27	42.53	34.02	0.96	4.08	16.26	21.3
Urban	42.05	48.96	52.02	56.36	52.94	6.91	3.06	4.34	14.31
**State-wise residence**									
EAG states	9.13	8.78	14.80	27.69	20.56	−0.35	6.02	12.89	18.56
Other states	39.21	44.17	49.80	62.53	54.60	4.96	5.63	12.73	23.32
**Religion**									
Hindu	25.26	27.14	32.56	45.55	38.08	1.88	5.42	12.99	20.29
Muslim	23.35	26.87	29.40	45.54	37.72	3.52	2.53	16.14	22.19
Others	37.22	40.30	39.98	55.95	48.65	3.08	−0.32	15.97	18.73
**Social group**									
Scheduled caste	20.31	22.06	27.95	46.20	37.68	1.75	5.89	18.25	25.89
Scheduled tribe	15.02	14.69	21.81	43.41	33.08	−0.33	7.12	21.6	28.39
Others	27.80	31.07	35.12	46.33	39.51	3.27	4.05	11.21	18.53
**Educational level**									
No Education	14.74	12.36	14.78	27.15	18.40	−2.38	2.42	12.37	12.41
Primary	31.83	28.12	29.54	39.17	34.87	−3.71	1.42	9.63	7.34
Secondary	50.21	46.67	49.44	52.13	51.25	−3.54	2.77	2.69	1.92
Higher	69.27	70.23	76.35	58.69	61.74	0.96	6.12	−17.66	−10.58
**Wealth Quintile**									
Poorest	9.94	10.12	13.05	27.03	20.22	0.18	2.93	13.98	17.09
Poorer	15.09	15.72	20.81	41.93	31.63	0.63	5.09	21.12	26.84
Middle	24.47	27.10	33.55	52.02	42.02	2.63	6.45	18.47	27.55
Richer	34.63	40.21	47.47	56.28	49.43	5.58	7.26	8.81	21.65
Richest	54.74	59.40	63.76	61.08	60.11	4.66	4.36	−2.68	6.34
**Maternal age**									
≤ 19	23.39	23.92	30.23	46.50	36.02	0.53	6.31	16.27	23.11
20–24	27.24	30.46	33.90	45.73	39.87	3.22	3.44	11.83	18.49
**Birth order**									
1	29.69	35.23	40.13	49.73	44.23	5.54	4.9	9.6	20.04
2	24.87	26.34	29.93	42.88	36.08	1.47	3.59	12.95	18.01
3+	18.21	14.36	16.96	33.27	22.62	−3.85	2.6	16.31	15.06
**Mass media exposure**									
No exposure	12.95	11.29	14.03	24.95	17.74	−1.66	2.74	10.92	12
Any exposure	39.02	40.49	39.48	51.88	47.46	1.47	−1.01	12.4	12.86
Total	25.53	27.63	32.35	45.98	38.46	2.1	4.72	13.63	20.45

^a^full ANC use for the pooled data

### Trends in the prevalence of SBA utilization by background characteristics

The highest increment in the proportion of young women who had SBA was seen during 2006–16 in all the categories. The utilization of SBA increased from 30 to 86% among rural women and from 68 to 94% among urban women between 1992 and 2016. In EAG states, the percentage of young mothers who had SBA rose from 23 to 83% during the study period, with the highest increment of 47% during 2006–16. Hindu women showed the highest increment in the use of SBA followed by Muslims with a rise of 53 and 44 percentage points respectively. The prevalence of SBA was lowest in young women belonging to ST social group, yet this group showed the largest increase of 63% between 1992 and 2016. The percentage of uneducated women who had SBA increased from 23% in NFHS-1 to 76% in NFHS-4. Young women belonging to the poorer wealth quintile showed the largest increment in SBA utilization with an absolute change of 63% between 1992 and 2016 followed by poorest (59%) and middle class (58%) women. It is clear from the pooled data that on average 65% of adolescent mothers used SBA and the percentage is 72 for adult young mothers. Women with second birth order showed the largest increment (51%) in SBA utilization during the study period. Between 1992 and 2016, the prevalence of SBA rose from 54 to 92% among women who had exposure to any media, whereas it increased from 23 to 75% among women unexposed to any kind of mass media. (Table 4).

**Table 4:** Percentage of young married women who had skilled birth attendance by demographic and socioeconomic characteristics, India, 1990–2016

Variables	Prevalence (%) of skilled birth attendance					Percentage change			
	NFHS-1	NFHS-2	NFHS-3	NFHS-4	Total ^a^	NFHS-1 to NFHS-2	NFHS-2 to NFHS-3	NFHS-3 to NFHS-4	NFHS-1 to NFHS-4
**Type of residence**									
Rural	29.78	37.92	44.73	86.50	64.79	8.14	6.81	41.77	56.72
Urban	68.39	73.72	74.10	93.50	85.03	5.33	0.38	19.4	25.11
**State-wise residence**									
EAG states	23.48	29.17	36.76	83.45	60.50	5.69	7.59	46.69	59.97
Other states	49.65	59.28	66.50	92.58	77.69	9.63	7.22	26.08	42.93
**Religion**									
Hindu	37.01	44.88	52.57	89.66	70.16	7.87	7.69	37.09	52.65
Muslim	37.02	42.44	44.02	80.99	64.58	5.42	1.58	36.97	43.97
Others	53.33	61.80	63.77	88.61	75.90	8.47	1.97	24.84	35.28
**Social group**									
Scheduled caste	30.45	40.71	49.16	88.14	69.94	10.26	8.45	38.98	57.69
Scheduled tribe	18.08	26.28	32.93	80.98	59.12	8.2	6.65	48.05	62.9
Others	41.56	49.16	55.57	89.49	71.01	7.6	6.41	33.92	47.93
**Educational level**									
No Education	23.14	26.65	32.00	75.70	42.86	3.51	5.35	43.7	52.56
Primary	48.68	48.52	49.42	82.56	66.92	−0.16	0.9	33.14	33.88
Secondary	69.64	68.51	70.97	92.58	86.50	−1.13	2.46	21.61	22.94
Higher	92.08	88.58	93.39	97.01	95.50	−3.5	4.81	3.62	4.93
**Wealth quintile**									
Poorest	15.54	21.70	25.80	74.84	51.92	6.16	4.1	49.04	59.3
Poorer	22.73	30.30	39.93	86.20	63.14	7.57	9.63	46.27	63.47
Middle	34.23	45.24	54.61	92.54	72.22	11.01	9.37	37.93	58.31
Richer	53.27	63.07	69.68	95.48	80.72	9.8	6.61	25.8	42.21
Richest	79.12	82.73	87.78	97.17	90.57	3.61	5.05	9.39	18.05
**Maternal age**									
≤ 19	35.35	42.26	50.29	88.31	65.04	6.91	8.03	38.02	52.96
20–24	39.66	47.44	52.68	88.21	72.16	7.78	5.24	35.53	48.55
**Birth order**									
1	45.42	57.40	63.23	91.92	78.23	11.98	5.83	28.69	46.5
2	33.94	40.65	47.11	85.20	66.00	6.71	6.46	38.09	51.26
3+	28.01	27.54	30.62	75.79	45.62	−0.47	3.08	45.17	47.78
**Mass media exposure**									
No exposure	22.57	26.49	31.97	75.28	46.32	3.92	5.48	43.31	52.71
Any exposure	22.57	59.92	59.34	91.88	79.63	37.35	−0.58	32.54	69.31
Total	37.75	45.20	51.67	88.24	69.54	7.45	6.47	36.57	50.49

^a^SBA use for the pooled data

### Determinants of maternal health care service utilization among young married women

Table 5 presents the results of pooled logistic regression for the utilization of selected maternal health care services by young married women. After adjusting for other covariates, the overall probability of young women practicing full ANC during their last pregnancy increased by 1.8 times (95% CI:1.68–1.98) between NFHS-1 and NFHS-4, whereas the probability of young women availing themselves of SBA grew by 14 times (95% CI:12.66–14.75) during the same period. Young women living in urban areas were 27% (95% CI:1.18–1.36) more probable to utilize full ANC and 87% (95% CI:1.71–2.04) more probable to have SBA than rural women. Compared to women residing in EAG states, women residing in other states had 4.7 times (95% CI:4.46–4.99) and 2.5 times (95% CI:2.30–2.62) higher odds of full ANC and SBA utilization, respectively. Muslim young women were 22% (95% CI:0.72–0.84) and 42% (95% CI:0.54–0.64) less likely to use full ANC and SBA respectively than their Hindu counterparts. Young women belonging to the ST social group were disadvantaged with 32% (95% CI:0.60–0.75) less probability, whereas women belonging to non-SC/ST social group were 14% (95% CI:1.06–1.22) more likely to avail themselves of SBA compared to SC women. The probability increased significantly with the economic level (that is, wealth quintile) and with the educational level of women, in both the unadjusted and adjusted models. After adjustment, young mothers belonging to richest wealth quintile were 2.3 times (95% CI:2.10–2.63) and 4.4 times (95% CI:3.86–4.98) more likely to use full ANC and SBA, respectively, than the poorest group. Compared to women with no education, women with higher education had 3.4 times (95% CI:3.04–3.84) and 4.5 times (95% CI:3.72–5.44) higher odds of full ANC and SBA utilization, respectively. Young women who gave birth during the upper age group (20–24 years) were more likely to use full ANC (Adjusted Odds Ratio (AOR):1.18; 95% CI:1.13–1.24) and SBA (AOR:1.2; 95% CI:1.14–1.27) compared to adolescent mothers. The odds for use of both the maternal health care services decreased with increasing birth order. Young women with birth order three and above had lower odds of full ANC (AOR:0.55; 95% CI:0.51–0.59) and SBA (AOR:0.41; 95% CI:0.38–0.44) utilization compared to women with first birth order. Women who had exposure to mass media were more likely to utilize full ANC (AOR:1.67; 95%CI:1.57–1.78) and SBA (AOR:1.30; 95%CI:1.23–1.38) than women who did not have any mass media exposure.

**Table 5:** Demographic and socioeconomic determinants of maternal health care utilization among young married women in India, 1992–2016

Variables	Full ANC		SBA	
	Unadjusted OR (95% CI)	Adjusted OR (95% CI)	Unadjusted OR (95% CI)	Adjusted OR (95% CI)
**Survey round**				
NFHS-1 (ref)	1.00	1.00	1.00	1.00
NFHS-2	1.11* (1.02–1.21)	0.98 (0.89–1.06)	1.36** (1.25–1.48)	1.35** (1.25–1.46)
NFHS-3	1.40** (1.29–1.51)	1.21** (1.10–1.32)	1.76** (1.60–1.92)	1.73** (1.60–1.89)
NFHS-4	2.48** (2.33–2.61)	1.83** (1.68–1.98)	12.38** (11.53–13.29)	13.66** (12.66–14.75)
**Type of residence**				
Rural (ref)	1.00	1.00	1.00	1.00
Urban	2.68** (2.51–2.86)	1.27** (1.18–1.36)	3.80** (3.51–4.11)	1.87** (1.71–2.04)
**State-wise residence**				
EAG states (ref)	1.00	1.00	1.00	1.00
Other states	5.48** (5.16–5.81)	4.71** (4.46–4.99)	2.70** (2.51–2.90)	2.46** (2.30–2.62)
**Religion**				
Hindu (ref)	1.00	1.00	1.00	1.00
Muslim	0.95 (0.88–1.03)	0.78** (0.72–0.84)	0.85** (0.79–0.93)	0.58** (0.54–0.64)
Others	1.59** (1.44–1.75)	0.81** (0.73–0.91)	1.62** (1.45–1.80)	0.98 (0.87–1.11)
**Social group**				
Scheduled caste (ref)	1.00	1.00	1.00	1.00
Scheduled tribe	0.73** (0.66–0.80)	1.00 (0.90–1.10)	0.57** (0.52–0.63)	0.68** (0.60–0.75)
Others	1.26** (1.18–1.33)	1.10* (1.03–1.17)	1.20** (1.13–1.27)	1.14** (1.06–1.22)
**Educational level**				
No Education (ref)	1.00	1.00	1.00	1.00
Primary	2.58** (2.41–2.77)	1.50** (1.39–1.61)	2.74** (2.58–2.92)	1.53** (1.43–1.64)
Secondary	5.60** (5.28–5.93)	2.15** (2.02–2.30)	7.84** (7.41–8.29)	2.25** (2.10–2.40)
Higher	11.27** (10.17–12.48)	3.42** (3.04–3.84)	29.23** (24.32–35.13)	4.50** (3.72–5.44)
**Wealth Quintile**				
Poorest (ref)	1.00	1.00	1.00	1.00
Poorer	1.73** (1.60–1.87)	1.15** (1.06–1.24)	1.56** (1.47–1.66)	1.27** (1.18–1.37)
Middle	2.98** (2.76–3.21)	1.44** (1.33–1.56)	2.52** (2.35–2.69)	1.72** (1.58–1.87)
Richer	4.64** (4.28–5.04)	1.66** (1.52–1.82)	4.57** (4.26–4.91)	2.43** (2.22–2.67)
Richest	8.76** (7.98–9.62)	2.35** (2.10–2.63)	12.46** (11.30–13.73)	4.38** (3.86–4.98)
**Maternal age**				
≤ 19 (ref)	1.00	1.00	1.00	1.00
20–24	1.27** (1.22–1.32)	1.18** (1.13–1.24)	1.31** (1.26–1.36)	1.20** (1.14–1.27)
**Birth order**				
1 (ref)	1.00	1.00	1.00	1.00
2	0.69** (0.66–0.72)	0.72** (0.69–0.76)	0.55** (0.52–0.57)	0.51** (0.48–0.54)
3+	0.35** (0.33–0.38)	0.55** (0.51–0.59)	0.28** (0.26–0.29)	0.41** (0.38–0.44)
**Mass media exposure**				
No exposure (ref)	1.00	1.00	1.00	1.00
Any exposure	4.57** (4.31–4.85)	1.67** (1.57–1.78)	4.29** (4.10–4.49)	1.30** (1.23–1.38)

** *p* < 0.01; *p < 0.05

## Discussion

This study has attempted to assess trends and determinants of ANC and SBA use among young married women in India over a period of more than two decades (1992–2016). The study revealed that although the utilization of full ANC is showing an upward trend since 1992, there exists a substantial proportion of young women who are not practicing full ANC during pregnancy. Moreover, utilization of full ANC is unacceptably low in EAG states as 72% of young mothers belonging to these states did not go for full ANC during 2015–2016. Also, a prevalence difference of more than 30% in full ANC use between EAG and non-EAG states remains consistent throughout the study period.

A remarkable increase in the proportion of young women who had SBA during birth was evident during 2006–2016, which may be attributed to a well-funded and well-publicized conditional cash transfer scheme named Janani Suraksha Yojana (JSY; translated as safe motherhood scheme) launched by the Government of India in 2005. JSY has led to an extensive and rapid increase in institutional deliveries, from 18% in 2008, to more than 80% 10 years later [33], with higher uptake of the incentive among younger women compared to the older ones [34].

This trend is also validated by the results from multivariate analyses. Lesser growth in the utilization of full ANC compared to SBA among young women could be due to several facts, such as limited knowledge and understanding about the importance of antenatal care, social restrictions for the young married women, and lack of cash incentives as in the case of SBA [35, 36]. The present study has also revealed several demographic and socioeconomic predictors of the utilization of maternal health care services among young mothers.

It is observed that urban women were more likely to use maternal health care services. This finding is in line with many other previous studies [11, 13, 16, 37–39]. Regarding state-wise residence, the present study revealed that there exists a prominent gap between EAG and Non-EAG states in the utilization of both the maternity care services among young women. Based on multivariate analysis, women belonging to Non-EAG states were almost 5 times and 2.5 times more likely to use full ANC and SBA, respectively, compared to women belonging to EAG states. To the authors’ knowledge, disparity in the use of maternal health care services between EAG and Non-EAG states has not been reported in the literature, however many studies have observed regional inequalities [12, 14, 16]. There could be many explanations for the low coverage of maternal health care services in EAG states. In India, there is a substantial difference in accessibility and availability of health care from public health facilities between EAG and Non-EAG states [40–42]. EAG states (mostly northern and central region) have a high proportion of the population below the poverty line and a high proportion of women not exposed to education and mass media [43]**.**

Furthermore, the utilization of maternity care services among young women is found to be influenced by their religion and social group. Several studies from India [11–13, 16, 44] and other countries [37, 38, 45, 46] have reported similar findings. The present study has found that Muslim women were less likely to opt for full ANC and SBA. Religion-specific beliefs and traditional practices of Muslim women may lead to lower use of maternity care services among them [47, 48]. Regarding the social group, young women belonging to other than SC/ST group were more likely to use both the maternal health care services, which could be due to lack of access to health care services as women of these social groups have a higher probability of living under adverse circumstances [49, 50]. Also, the low coverage of maternal health care services among young Muslim and SC/ST women could be linked to their lower autonomy and lower socioeconomic status [47, 50, 51].

The educational status turned out to be a potent determinant of ANC and SBA utilization among young mothers A sharp increase in the odds of the utilization of both the maternal health care services was observed as we move from uneducated women to higher educated women. Many previous studies conducted in India [11–13, 16, 52] and other developing countries [53–55] have found similar results. Educated women have the capability to access health care information and are more aware of the negative consequences of not practicing maternity care services. Moreover, higher education may empower women to make proper decisions for their health and to use health care inputs accordingly [53, 56].

The present study has also found a significant disparity in maternal health care service utilization across different economic groups. Young women belonging to wealthier households were more likely to practice maternal health care than those who are from poor households. This rich-poor gap is consistent with the findings of many other studies from India [11–13, 16, 57, 58] and elsewhere [30, 53, 55]. Poor young women are often turned out to be uneducated, unemployed, and detached from social networks; they are thus more difficult to be reached by maternity care programs, and they tend to underestimate the importance of maternal health care services and therefore prioritize spending their limited resources on daily basic needs over maternal health care [59, 60].

Our study revealed that adolescent mothers were less likely to use full ANC and SBA than adult young mothers. This finding is supported by a study conducted in Kenya on young women [54]. Knowledge and experience of elder women encourage them to get maternity and childbirth care [61]. Additionally, older young women have higher decision making autonomy, which has a positive association with greater use of maternal health care services [51, 62]. Therefore, delaying childbearing of young women would be beneficial for greater coverage of maternity care services.

Concerning birth order, it is observed that the likelihood of using full ANC and SBA decreases significantly with the increase in birth order. Several other studies have reported similar finding [12, 13, 44, 63]. Primiparous women may have fear of first pregnancy and are more afraid of complications and difficulties during delivery. Whereas, during higher-order births, women may have developed self-confidence as they have prior experience of birth/births, which makes them less likely to seek maternal health care services [63, 64]. The other reasons may be resource constraints due to having more children and prior bad health facility experiences [63].

Finally, mass media exposure was found to have a significant positive association with maternity care service utilization. Young mothers exposed to any kind of mass media were more likely to opt for full ANC and SBA. This result concurs with the findings from various other studies [11, 12, 16, 65]. Mass media is an important source of information on health and existing health care programs or policies. Women, who are exposed to mass media, may have a better understanding of maternal health complications and the importance of antenatal care and skilled birth attendance during delivery. Women’s exposure to mass media may also be associated with other factors like higher wealth quintile, higher education, and urban residence, all of which are positively associated with an increased likelihood of utilizing maternal health care services.

The government of India has launched several programs to reduce inequity in health care services and improve reproductive, maternal, new-born and child health outcomes. These programs included the National Rural Health Mission (2005), the National Urban Health Mission (2008), and the Reproductive, Maternal, Newborn, Child, and Adolescent Health (RMNCH+A) Strategy. The RMNCH+A strategy was introduced in 2013 to strengthen maternity care services especially in the poor-performing regions of the country [66]. It is evident from this study that there is a quite significant and persistent gap in utilization of full ANC and SBA between EAG and other states in India among young mothers. Also, the coverage of full ANC remained fairly low, even during 2015–16. These findings question the effective implementation of ongoing programs and policies. Therefore, our study recommends close monitoring of the ongoing programs and focused initiatives to reach out to the young women who are in need of essential maternity care services. To address the low coverage of full ANC, we suggest that the government should either launch new cash initiatives covering ANC services or include ANC services under existing schemes such as the newly launched National Health Protection Scheme under Ayushman Bharat Yojana.

### Strengths and limitations

The main strength of our study is that it is based on the four waves of a nationally representative survey, which covers a time period of more than two decades (1992–2016). Consequently, the findings of this study can be generalized to all women aged 15 to 24 years in India. The standardized methodology used by the DHS also allows comparisons with similar data across different countries. Despite these strengths, the findings of this study should not be interpreted without acknowledging its limitations. Some of the potential limitations of our study include recall bias, reporting bias, and non-availability of required information in some or all of the survey rounds. The responses on age, antenatal care, skilled birth attendance were self-reported by women and hence are prone to recall and reporting bias. During multivariate analysis, important variables representing women’s decision-making autonomy were excluded due to lack of data on these variables. Since the information on maternal health care indicators was available only for pregnancies that resulted in a live birth, we were not able to analyse the section of the population with adverse outcomes i.e. miscarriage, abortion or stillbirth. Also, there was no data on the quality and accessibility of maternal health care services which could have given a more informed idea on the adequacy of maternity care.

## Conclusions

In India, the utilization of full ANC remained unjustifiably low among young women between 1992 and 2016. Although the proportion of young mothers who availed themselves of SBA increased substantially during the period, the improvement appeared inequitable. Since inadequate use of these maternity care services could lead to fatal health outcomes in adolescent and adult young mothers, there is a pressing need to develop targeted policies. Increased efforts should be made to address low coverage of these services among underprivileged segments of the population such as poor households, rural residents, Muslim and SC/ST women. This study highlights a wide and persistent gap between EAG and non-EAG states in the utilization of maternal health care services among young mothers. Consequently, it is necessary to ensure effective implementation of ongoing programs by improving public health network and the quality of physical and human infrastructure in EAG states. Lastly, the government should prioritize young women’s education and their accessibility to mass media, which may help in increasing knowledge and awareness about the importance of maternity care services.

## Data Availability

The dataset analyzed cannot be made publicly available by us as it belongs to the DHS program, but it can be accessed from the following link after acquiring permission from Measure DHS: https://www.dhsprogram.com/data/available-datasets.cfm

## Abbreviations

MMR: Maternal Mortality Ratio
EAG: Empowered Action Group
ANC: Antenatal Care
SBA: Skilled Birth Attendance
NFHS: National Family Health Survey
DHS: Demographic and Health Survey
MoHFW: Ministry of Health and Family Welfare
SC: Scheduled Caste
ST: Scheduled Tribes
AOR: Adjusted Odds Ratio

## References

[1] World Health Organization. Trends in maternal mortality 2000 to 2017: estimates by WHO, UNICEF: UNFPA, World Bank Group and the United Nations Population Division, Geneva; 2019. Retrieved April, 1, 2020

[2] Alkema L, Chou D, Hogan D, Zhang S, Moller AB, Gemmill A (2016). Global, regional, and national levels and trends in maternal mortality between 1990 and 2015, with scenario-based projections to 2030: a systematic analysis by the UN maternal mortality estimation inter-agency group. Lancet.

[3] Sample Registration System. SPECIAL BULLETIN ON MATERNAL MORTALITY IN INDIA 2015–17. Available at: http://censusindia.gov.in/vital_statistics/SRS_Bulletins/MMR_Bulletin-2015-17.pdf. Last accessed on 16 Feb 2020.

[4] Bauserman M, Lokangaka A, Thorsten V, Tshefu A, Goudar SS, Esamai F (2015). Risk factors for maternal death and trends in maternal mortality in low-and middle-income countries: a prospective longitudinal cohort analysis. Reprod Health..

[5] Bhutta ZA, Das JK, Rizvi A, Gaffey MF, Walker N, Horton S (2013). Evidence-based interventions for improvement of maternal and child nutrition: what can be done and at what cost?. Lancet.

[6] Graham WJ, Bell JS, Bullough CH (2001). Can skilled attendance at delivery reduce maternal mortality in developing countries?. Safe motherhood strategies: a review of the evidence.

[7] UNFPA (2005). University of Aberdeen: Maternal Mortality Update 2004: Delivering into Good Hands.

[8] World Health Organization. WHO recommendations on antenatal care for a positive pregnancy experience. 2016. Available at. https://apps.who.int/iris/bitstream/handle/10665/250796/9789241549912-eng.pdf. Accessed 4 Feb 2021. 28079998

[9] World Health Organization. Making pregnancy safer: the critical role of the skilled attendant: a joint statement by WHO, ICM and FIGO. 2004. Available at https://apps.who.int/iris/bitstream/handle/10665/42955/9241591692.pdf. Accessed 4 Feb 2021.

[10] International Institute for Population Sciences (IIPS) and ICF (2017). National Family Health Survey (NFHS-4), 2015–16: India.

[11] Pathak PK, Singh A, Subramanian SV (2010). Economic inequalities in maternal health care: prenatal care and skilled birth attendance in India, 1992–2006. PLoS One.

[12] Singh PK, Rai RK, Alagarajan M, Singh L (2012). Determinants of maternity care services utilization among married adolescents in rural India. PLoS One.

[13] Kumar G, Choudhary TS, Srivastava A, Upadhyay RP, Taneja S, Bahl R (2019). Utilisation, equity and determinants of full antenatal care in India: analysis from the National Family Health Survey 4. BMC Pregnancy Childbirth.

[14] Ogbo FA, Dhami MV, Ude EM, Senanayake P, Osuagwu UL, Awosemo AO (2019). Enablers and barriers to the utilization of antenatal care services in India. Int J Environ Res Public Health.

[15] Singh R, Neogi SB, Hazra A, Irani L, Ruducha J, Ahmad D (2019). Utilization of maternal health services and its determinants: a cross-sectional study among women in rural Uttar Pradesh, India. J Health Popul Nutr.

[16] Paul P, Chouhan P (2020). Socio-demographic factors influencing utilization of maternal health care services in India. Clin Epidemiol Global Health..

[17] United Nations Department of Economic and Social Affairs (UNDESA). Definition of youth. http://www.unesco.org/new/en/social-and-human-sciences/themes/youth/youth-definition/. Last accessed on 16 Jan 2021.

[18] Shah IH, Åhman E (2012). Unsafe abortion differentials in 2008 by age and developing country region: high burden among young women. Reprod Health Matters.

[19] MacQuarrie K, Mallick L, Allen C (2017). Sexual and reproductive health in early and later adolescence: DHS data on Yourth age 10–19.

[20] UNICEF, 2020*.* Available at: https://data.unicef.org/topic/hivaids/adolescents-young-people/#:~:text=HIV%20in%20adolescents,of%20new%20adult%20HIV%20infections*.* Last accessed on 17 Dec 2020.

[21] Conde-Agudelo A, Belizán JM, Lammers C (2005). Maternal-perinatal morbidity and mortality associated with adolescent pregnancy in Latin America: cross-sectional study. Am J Obstet Gynecol.

[22] Vogel JP, Souza JP, Mori R, Morisaki N, Lumbiganon P, Laopaiboon M (2014). Maternal complications and perinatal mortality: findings of the World Health Organization multicountry survey on maternal and newborn health. BJOG Int J Obstet Gynaecol.

[23] World Health Organisation (2014). Global Health Estimates 2013 Summary tables: DALYs, YLLs and YLDs by cause, age and sex by WHO regional group and World Bank income classification, 2000–2012 (provisional estimates).

[24] Central Statistics Office, Ministry of Statistics and Programme Implementation, Government of India (Social Statistics Division) (2017). Youth in India. Available at http://mospi.nic.in/sites/default/files/publication_reports/Youth_in_India-2017.pdf. Accessed 4 Feb 2021.

[25] Godha D, Hotchkiss DR, Gage AJ (2013). Association between child marriage and reproductive health outcomes and service utilization: a multi-country study from South Asia. J Adolesc Health.

[26] Paul P, Chouhan P (2019). Association between child marriage and utilization of maternal health care services in India: evidence from a nationally representative cross-sectional survey. Midwifery.

[27] International Institute for Population Sciences (1995). National Family Health Survey India, 1992–93 NFHS-1.

[28] International Institute for Population Sciences, ORC Macro (2000). National Family Health Survey India, 1998–99 NFHS-2.

[29] International Institute for Population Sciences, ORC Macro (2007). National family health survey India, 2005–06 NFHS-3.

[30] Banke-Thomas OE, Banke-Thomas AO, Ameh CA (2017). Factors influencing utilisation of maternal health services by adolescent mothers in low-and middle-income countries: a systematic review. BMC Pregnancy Childbirth.

[31] Ram F, Roy TK (2004). Comparability issues in large sample surveys-some observations. Population, health and development in India-Changing Perspectives, International Institute for Population Sciences, Mumbai.

[32] Mishra V, Roy TK, Retherford RD (2004). Sex differentials in childhood feeding, health care, and nutritional status in India. Popul Dev Rev.

[33] Anand R, Singh R, Srivastava R (2016). Impact of Janani Suraksha Yojana on institutional delivery rate, incidence of rupture uterus and feto-maternal outcome related to uterine rupture. Int J Reprod Contracept Obstet Gynecol.

[34] Lim SS, Dandona L, Hoisington JA, James SL, Hogan MC, Gakidou E (2010). India's Janani Suraksha Yojana, a conditional cash transfer programme to increase births in health facilities: an impact evaluation. Lancet.

[35] Shahabuddin A, Nöstlinger C, Delvaux T, Sarker M, Delamou A, Bardají A (2017). Exploring maternal health care-seeking behavior of married adolescent girls in Bangladesh: a social-ecological approach. PLoS One.

[36] Gupta M. Life course perspectives on women’s autonomy and health outcomes. Health Trans Rev. 1996;6:213–31.

[37] Say L, Raine R (2007). A systematic review of inequalities in the use of maternal health care in developing countries: examining the scale of the problem and the importance of context. Bull World Health Organ.

[38] Tarekegn SM, Lieberman LS, Giedraitis V (2014). Determinants of maternal health service utilization in Ethiopia: analysis of the 2011 Ethiopian demographic and health survey. BMC Pregnancy Childbirth.

[39] Rutaremwa G, Wandera SO, Jhamba T, Akiror E, Kiconco A (2015). Determinants of maternal health services utilization in Uganda. BMC Health Serv Res.

[40] Rani M, Bonu S, Harvey S (2008). Differentials in the quality of antenatal care in India. Int J Qual Health Care.

[41] Kumar V, Singh P (2016). Access to healthcare among the empowered action group (EAG) states of India: current status and impeding factors. Natl Med J India.

[42] Sanasam DC (2020). Maternal reproductive health: a comparison between India and empowered action group states. Urban Health Risk and Resilience in Asian Cities.

[43] NITI Ayog : RBI Handbook of Statistics on Indian Economy 2015–16. Available at: https://rbidocs.rbi.org.in/rdocs/Publications/PDFs/154T_HB15092019609736EE47614B23BFD377A47FFC1A5D.PDF. Last accessed on 6 June 2020.

[44] Ali B, Chauhan S (2020). Inequalities in the utilisation of maternal health care in rural India: evidences from national family health survey III & IV. BMC Public Health.

[45] Gyimah SO, Takyi BK, Addai I (2006). Challenges to the reproductive-health needs of African women: on religion and maternal health utilization in Ghana. Soc Sci Med.

[46] Målqvist M, Lincetto O, Du NH, Burgess C, Hoa DTP (2013). Maternal health care utilization in Viet Nam: increasing ethnic inequity. Bull World Health Organ.

[47] Basant R (2007). Social, economic and educational conditions of Indian muslims. Econ Politic Wkly..

[48] Hazarika I (2011). Factors that determine the use of skilled care during delivery in India: implications for achievement of MDG-5 targets. Matern Child Health J.

[49] Deshpande A (2000). Does caste still define disparity? A look at inequality in Kerala, India. Am Econ Rev.

[50] Nayar KR (2007). Social exclusion, caste & health: a review based on the social determinants framework. Indian J Med Res.

[51] Mistry R, Galal O, Lu M (2009). Women's autonomy and pregnancy care in rural India: a contextual analysis. Soc Sci Med.

[52] Barman B, Saha J, Chouhan P (2020). Impact of education on the utilization of maternal health care services: an investigation from National Family Health Survey (2015–16) in India. Child Youth Serv Rev.

[53] Ahmed S, Creanga AA, Gillespie DG, Tsui AO (2010). Economic status, education and empowerment: implications for maternal health service utilization in developing countries. PLoS One.

[54] Ochako R, Fotso JC, Ikamari L, Khasakhala A (2011). Utilization of maternal health services among young women in Kenya: insights from the Kenya demographic and health survey, 2003. BMC Pregnancy Childbirth.

[55] Gebre E, Worku A, Bukola F (2018). Inequities in maternal health services utilization in Ethiopia 2000–2016: magnitude, trends, and determinants. Reprod Health.

[56] Jejeebhoy SJ (1995). Women's education, autonomy, and reproductive behaviour: experience from developing countries. OUP Catalogue.

[57] Mohanty SK, Pathak PK (2009). Rich-poor gap in utilization of reproductive and child health services in India, 1992-2005. J Biosoc Sci.

[58] Kesterton AJ, Cleland J, Sloggett A, Ronsmans C (2010). Institutional delivery in rural India: the relative importance of accessibility and economic status. BMC Pregnancy Childbirth.

[59] Rani M, Lule E (2004). Exploring the socioeconomic dimension of adolescent reproductive health: a multicountry analysis. Intl Fam Plan Perspect..

[60] Rai RK, Tulchinsky TH (2015). Addressing the sluggish progress in reducing maternal mortality in India. Asia Pac J Public Health.

[61] Magadi MA, Agwanda AO, Obare FO (2007). A comparative analysis of the use of maternal health services between teenagers and older mothers in sub-Saharan Africa: evidence from demographic and health surveys (DHS). Soc Sci Med.

[62] Haque SE, Rahman M, Mostofa MG, Zahan MS (2012). Reproductive health care utilization among young mothers in Bangladesh: does autonomy matter?. Womens Health Issues.

[63] Chakraborty N, Islam MA, Chowdhury RI, Bari W, Akhter HH (2003). Determinants of the use of maternal health services in rural Bangladesh. Health Promot Int.

[64] Fotso JC, Ezeh A, Oronje R (2008). Provision and use of maternal health services among urban poor women in Kenya: what do we know and what can we do?. J Urban Health.

[65] Acharya D, Khanal V, Singh JK, Adhikari M, Gautam S (2015). Impact of mass media on the utilization of antenatal care services among women of rural community in Nepal. BMC Res Notes.

[66] Ministry of Health and Family Welfare. A strategic approach to reproductive, maternal, newborn, child and adolescent health (RMNCH+ A) in India. 2013. Available at https://nhm.gov.in/images/pdf/RMNCH+A/RMNCH+A_Strategy.pdf. Accessed 4 Feb 2021.

